# TGF-β Induces the Secretion of Extracellular Vesicles Enriched with CD39 and CD73 from Cervical Cancer Cells

**DOI:** 10.3390/ijms26062413

**Published:** 2025-03-07

**Authors:** Gabriela Molina-Castillo, Alberto Monroy-García, Rosario García-Rocha, Benny Weiss-Steider, Juan José Montesinos-Montesinos, Jorge Hernández-Montes, Christian Azucena Don-López, Marta Elena Castro-Manrreza, María Luisa Escobar-Sánchez, María de Lourdes Mora-García

**Affiliations:** 1 Laboratorio de Inmunobiología, Unidad de Investigación en Diferenciación Celular y Cáncer-UMIEZ, FES-Zaragoza, Universidad Nacional Autónoma de México, Ciudad de México 09230, Mexico; gmolina2509@comunidad.unam.mx (G.M.-C.); rgrbiologia@hotmail.com (R.G.-R.); bennyweiss@hotmail.com (B.W.-S.); jgehdzmts@gmail.com (J.H.-M.); damostwanted060993@live.com.mx (C.A.D.-L.); 2 Programa de Posgrado en Ciencias Biológicas, UNAM, Ciudad de México 09230, Mexico; 3 Laboratorio de Inmunología y Cáncer, Unidad de Investigación Médica en Enfermedades Oncológicas, Hospital de Oncología, CMN SXXI, Instituto Mexicano del Seguro Social, Ciudad de México 06720, Mexico; 4 Laboratorio de Células Troncales Mesenquimales, Unidad de Investigación Médica en Enfermedades Oncológicas, CMN SXXI, Instituto Mexicano del Seguro Social, Ciudad de México 06720, Mexico; montesinosster@gmail.com; 5 Laboratorio de Inmunología y Células Madre, Unidad Multidisciplinaria de Investigación Experimental Zaragoza, FES Zaragoza, Universidad Nacional Autónoma de México, Ciudad de México 09230, Mexico; elmar_ca@yahoo.com.mx; 6 Laboratorio de Microscopía Electrónica, Departamento de Biología Celular, Facultad de Ciencias, Universidad Nacional Autónoma de México, Ciudad Universitaria, Ciudad de México 04510, Mexico; escobarluisa@ciencias.unam.mx

**Keywords:** TGF-β, CD39, CD73, cervical cancer cells, extracellular vesicles

## Abstract

The presence of TGF-β in the tumor microenvironment of cervical cancer (CC) is important for tumor progression. In this study, we analyzed the effect of TGF-β on the expression of the ectonucleotidases CD39 and CD73, which are involved in the generation of adenosine (Ado), in CC cells and in extracellular vesicles (EVs) secreted by these cells. Treatment of HeLa and CaSki cells for 72 h with recombinant human TGF-β increased the expression of CD39 and CD73 by 20 and 30% and by 40 and 100%, respectively. The addition of SB505124, an inhibitor of the TGF-β1 receptor, or GW4869, an inhibitor of exosome formation and release, reduced the expression and release of both ectonucleotidases in CC cells. Furthermore, TGF-β promoted the secretion of medium-large EVs (>130 nm) in HeLa cells (HeLa + TGF-β/EVs) and CaSki cells (CaSki + TGF-β/EVs), which increased the expression of CD39 (>20%) and CD73 (>60%), and EVs obtained from cells treated with TGF-β had a greater capacity to generate Ado than did EVs obtained from cells cultured in the absence of this factor (HeLa/EVs and CaSki/EVs). These findings suggest that the production of TGF-β in the CC TME can promote neoplastic progression through the secretion of EVs enriched with CD39 and CD73. Therefore, the inhibition of CD39+ CD73+ EVs could be a strategy for the treatment of CC.

## 1. Introduction

Among all types of cancer, CC ranks fourth in terms of incidence and mortality worldwide. In 2022, approximately 660,000 new cases and more than 348,000 deaths from CC were registered. Data from the World Health Organization indicate that nearly 90% of deaths associated with CC occur in developing countries [1].

Infections caused by high-risk human papillomaviruses (HR-HPVs) such as the HPV-16 and HPV-18 genotypes, which are present in approximately 60 and 15% of invasive CC cases, respectively, are considered etiological factors [2], since persistent HR-HPV infection reportedly promotes immune evasion and suppression in tumor cells via a range of strategies, such as (a) decreasing the expression of chemokines (CXCL14), Toll-like receptors and adhesion molecules (E-cadherin) on host cells to disrupt the recruitment of dendritic cells, T cells, natural killer (NK) cells, and Langerhans cells, at the site of infection [3,4]; (b) altering the expression of MHC class I molecules (MHC-I), the subunits of the immunoproteasome and the transporter associated with antigen processing (TAP) in infected cells, which leads to viral escape from immunosurveillance and increases the risk of lesion progression [5]; (c) favoring an immunosuppressive tumor microenvironment (TME), which is characterized by a Th2 immunophenotype, with a significant decrease in the ratio of CD8+/CD4+ T cells [6]; and (d) enabling the increased expression of immune checkpoint genes, such as programmed cell death 1 (PD-1), lymphocyte activator 3 (LAG3) and hepatitis A virus cell receptor-2 (HAVR2), in T cells [7].

On the other hand, it has been reported that the generation of large amounts of Ado in the TME contributes significantly to the suppression of the immune response in gynecological cancers, including CC [8]. This nucleoside is generated through the adenosinergic pathway, in which extracellular adenosine triphosphate (ATP) is sequentially hydrolyzed to adenosine diphosphate (ADP) and adenosine monophosphate (AMP) by the enzyme ectonucleoside triphosphate diphosphohydrolase-1 (CD39). The resulting AMP is subsequently hydrolyzed to Ado by the enzyme 5′-nucleotidase (CD73) [9]. The signaling activities of Ado in target cells are carried out by four subtypes of adenosine receptors (A1R, A2AR, A2BR, and A3R) coupled to G proteins in the membrane [10]. The increased expression of CD39 and CD73 in various types of cancer is positively correlated with increased expression of hypoxia-inducible factor-1 (HIF-1) and the presence of some proinflammatory cytokines, such as tumor necrosis factor (TNF-α), interleukin-2 (IL-2), and interleukin-6 (IL-6), in the TME [11,12]. However, several studies have reported that an increase in the expression of CD39 and CD73 in tumor cells can be induced through TGF-β activity [13,14]. The production of TGF-β1 during the development of CC has been associated with the expression of the HR-HPV oncogenes E6 and E7, which induce the activation of the human TGF-β1 promoter [15,16]. In previous studies, we reported that HR-HPV-infected CC tumor cells constitutively produce TGF-β1, which is important for inducing and maintaining CD73 expression [17].

The secretion of EVs by different types of cells, including tumor cells, can induce the formation of an immunosuppressive microenvironment through the generation of Ado [18]. EVs are a heterogeneous population of membrane vesicles released naturally by cells; these vesicles are delimited by a lipid bilayer and do not have the ability to replicate. EVs are commonly classified by their size into small (sEVs < 200 nm) and medium-large (m/lEVs > 200 nm) EVs [19]. According to their biogenesis process, EVs are classified as exosomes, microvesicles, ectosomes, oncosomes, or apoptotic bodies [19]. Exosomes are formed as intraluminal vesicles (ILVs) through the internal budding of endosomal membranes in multivesicular bodies (MVBs) and have diameters between 40 and 130 nm [20]. Microvesicles, whose size varies between 150 and 1000 nm, are generated through shoots and fusions of the plasma membrane in the extracellular space [21]. EVs secreted by tumor cells have been implicated in various processes related to tumor progression, including the proliferation, migration, and metastasis of tumor cells; drug resistance; and suppression of the antitumor immune response. EVs can contain various types of molecules, such as nucleic acids (e.g., DNA, mRNA, miRNA, and cRNA), lipids, metabolites, signaling molecules, and cell surface receptors [22,23]. However, the factors that regulate the secretion and function of EVs have not yet been fully elucidated. Several studies have revealed that signaling through inflammatory pathways is the main regulator of EV biogenesis during the carcinogenesis process [24,25]. In this context, it has been proposed that because of its dual, proinflammatory/anti-inflammatory nature, TGF-β-mediated signaling can participate in the generation and loading of EVs [26,27,28]. Considering these findings and the association of the production of TGF-β with the development of CC, the aim of this study was to analyze the effect of TGF-β on the expression of CD39 and CD73 ectonucleotidases in CC cells and EVs secreted by these cells.

## 2. Results

### 2.1. TGF-β Induced the Expression of CD39 and CD73 in CC Cells

**Figure 1:**
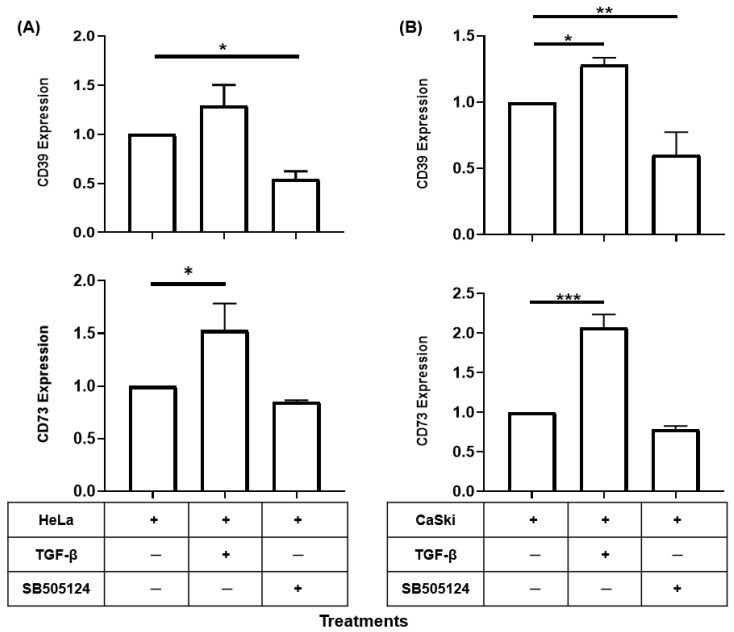
TGF-β induces and maintains the expression of CD39 and CD73 in CC cells. HeLa and CaSki cells, 5 × 10^4^ each, were cultured in the presence or absence of 20 ng/mL rh-TGF-β or in the presence of 25 µM SB505124, a selective inhibitor of TGF-β1 receptors. After 72 h, the expression of CD39 and CD73 on the cell membrane was analyzed via flow cytometry. The baseline expression of these molecules in each CC cell line was normalized to 1. The expression levels of CD39 and CD73 in HeLa (**A**) and CaSki (**B**) cells cultured under different treatments are shown. The statistical significance was calculated using the one-way ANOVA followed by Dunnett’s post hoc test for multiple comparisons. Representative data from three independent experiments ± the standard error of the mean (SEM) are shown. * *p* < 0.05; ** *p* < 0.01; *** *p* < 0.001.

The induction of CD39 and CD73 expression in different tumors has been reported to be related to TGF-β signaling [13,14,15]. To analyze whether TGF-β induces the expression of CD39 and CD73 in CC cells, 5 × 10^4^ HeLa or CaSki cells were cultured in the presence of 20 ng/mL rh-TGF-β, as previously reported [17]. After 72 h, the expression of CD39 and CD73 was analyzed via flow cytometry. The baseline expression of these molecules in each CC cell line was normalized to 1. The addition of rh-TGF-β to HeLa and CaSki cell cultures strongly increased the expression of CD39 and CD73, with values of 1.30 ± 0.20 and 1.56 ± 0.28 and of 1.29 ± 0.06 and 2.07 ± 0.06, respectively (Figure 1A,B). The addition of SB505124, a selective inhibitor of the TGF-β1 receptor, to cultures of HeLa and CaSki cells reduced the expression of CD39 and CD73, with values of 0.545 ± 0.139 and 0.87 ± 0.012 and of 0.604 ± 0.169 and 0.787 ± 0.04, respectively (Figure 1A,B). These results indicate that TGF-β is an important factor in inducing and maintaining the expression of CD39 and CD73 in CC cells.

### 2.2. GW4869 Abrogated the Increase in the Release of CD39 and CD73 Induced by TGF-β in CC Cells

**Figure 2:**
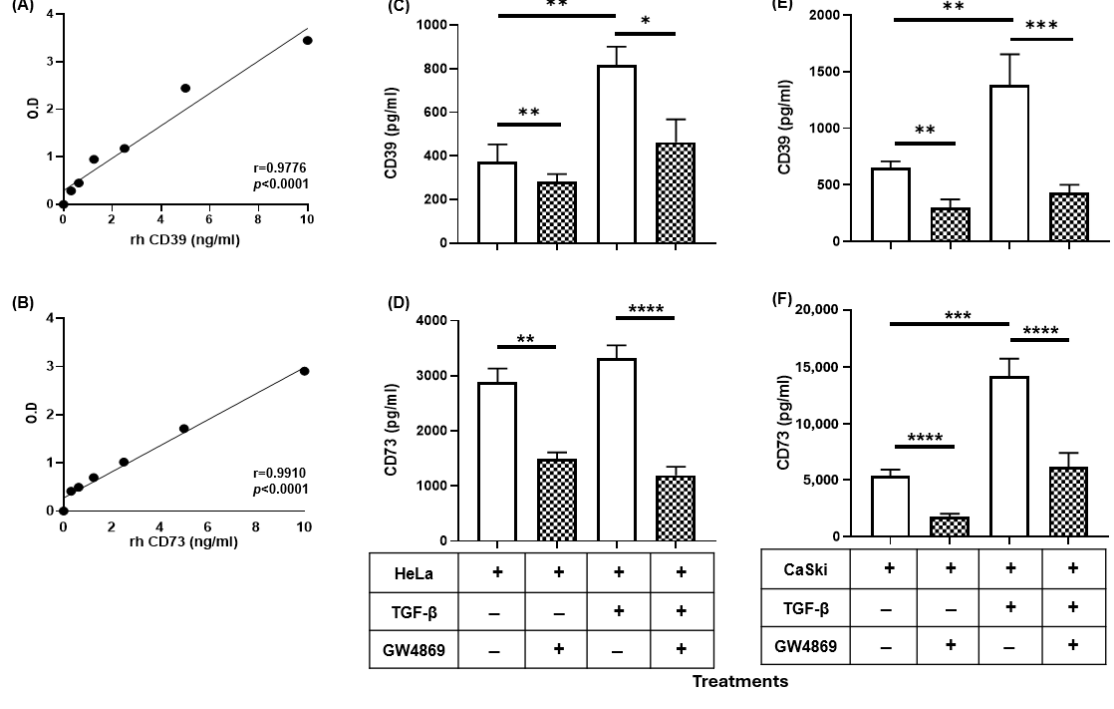
CD39 and CD73 levels in CC cell supernatants. HeLa and CaSki cells (1 × 10^6^) were cultured for 72 h in the presence or absence of 20 ng/mL TGF-β and in the presence or absence of 20 µM GW4869, an inhibitor of exosome formation and release. The concentrations of CD39 and CD73 in the supernatants of HeLa and CaSki cells were evaluated via ELISA using standard curves generated with different known concentrations (0–10 ng/mL) of rh-CD39 (**A**) and rh-CD73 (**B**). The concentrations of CD39 and CD73 in the supernatants of HeLa (**C**,**D**) and CaSki (**E**,**F**) cells were determined by interpolating the unknown values from the standard curves. The statistical significance was calculated using the one-way ANOVA followed by Sidak’s post hoc test for multiple comparisons. Representative data from three independent experiments ± SEM are shown. * *p* < 0.05; ** *p* < 0.01; *** *p* < 0.001; **** *p* < 0.0001.

The release of CD39 and CD73 in the TME has been proposed as a mechanism by which tumor cells contribute to the suppression of the immune response through the generation of Ado [18]. To analyze the effect of TGF-β on the release of CD39 and CD73 from CC cells, 2.5 × 10^5^ HeLa or CaSki cells were cultured for 72 h in the absence or presence of 20 ng/mL rh-TGF-β in serum-free medium. The levels of these ectonucleotidases in the supernatants were evaluated via enzyme-linked immunosorbent assay (ELISA). Standard curves were generated using different concentrations (0.5–10 ng/mL) of the recombinant human enzymes CD39 (rh-CD39) (Figure 2A) and CD73 (rh-CD73) (Figure 2B). The amounts of CD39 and CD73 in the supernatants of HeLa and CaSki cells cultured in the presence of rh-TGF-β were greater than those in the supernatants of cells cultured in the absence of this cytokine. In the supernatants of HeLa cells cultured in the absence or presence of rh-TGF-β, the concentrations of CD39 and CD73 were 463 ± 108 and 831 ± 85 pg/mL and 2882 ± 252 and 3348 ± 248 pg/mL, respectively (Figure 2C,D, white bars). In the supernatants of CaSki cells treated under the same conditions, the concentrations of CD39 and CD73 were 816 ± 162 and 1002 ± 213 pg/mL and 5295 ± 670 and 14,223 ± 1764 pg/mL, respectively (Figure 2E,F, white bars).

To determine whether the CD39 and CD73 ectoenzymes detected in the supernatants of CC cells were EVs, we evaluated the effect of GW4869, a known inhibitor of the formation and release of exosomes. For this purpose, HeLa and CaSki cells were cultured in the presence of 20 µM GW4869 in the absence or presence of 20 ng/mL rh-TGF-β. Interestingly, the addition of GW4869 significantly reduced the release of CD39 and CD73 induced by TGF-β in HeLa and CaSki cell cultures (Figure 2, boxed bars). The concentrations of CD39 and CD73 in the supernatants of HeLa cells cultured in the presence of GW4869 and in the absence or presence of rh-TGF-β were 276 ± 57 and 462 ± 114 pg/mL and 1490 ± 148 and 1190 ± 112 pg/mL, respectively (Figure 2C,D). The concentrations of CD39 and CD73 in the supernatants of CaSki cells treated under the same conditions were 298 ± 85 and 359 ± 90 pg/mL and 1764 ± 294 and 6135 ± 1470 pg/mL, respectively (Figure 2E,F, boxed bars). Taken together, these results indicate that TGF-β induces the release of the ectonucleotidases CD39 and CD73 through the secretion of EVs.

### 2.3. TGF-β Increased the Release of CD39- and CD73-Positive EVs from CC Cells

Recent studies have reported that TGF-β promotes the generation and loading of EVs [26,28]. Therefore, we analyzed the effects of TGF-β on the release of CD39- and CD73-positive EVs from CC cells cultured under baseline conditions (HeLa/EV and CaSki/EV) and in the presence of rh-TGF-β (HeLa + TGF-β/EVs and CaSki + TGF-β/EVs). For this purpose, 1 × 10^7^ HeLa or CaSki cells were cultured for 72 h in the presence or absence of 20 ng/mL rh-TGF-β, after which EVs were obtained from the supernatants.

**Figure 3:**
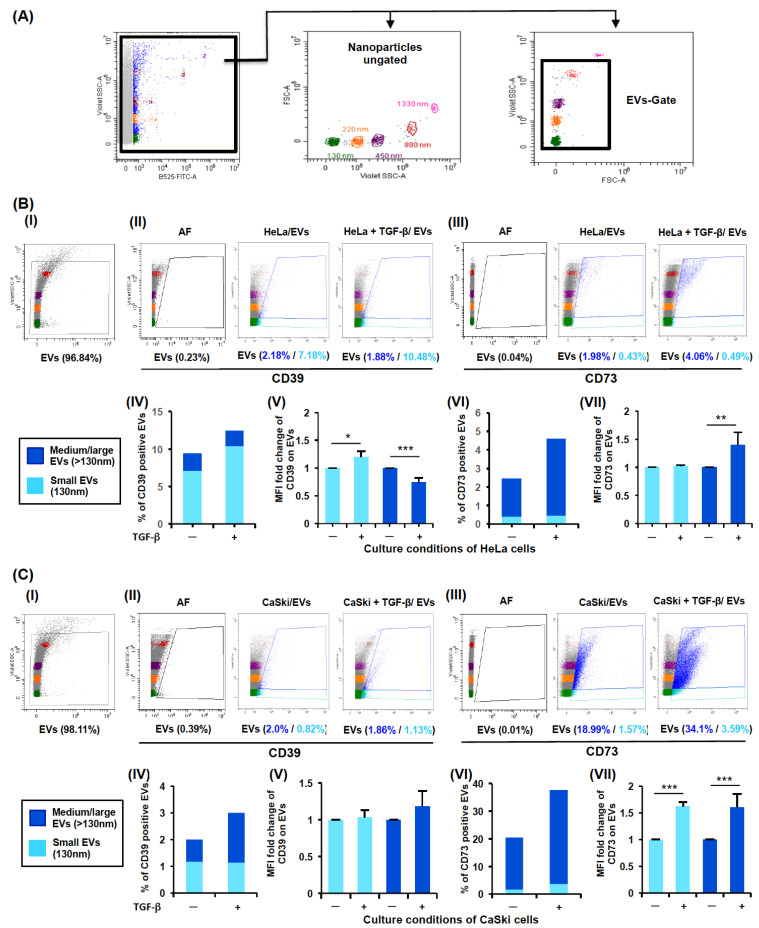
Effect of TGF-β on the secretion of CD39+ and CD73+ EVs by CC cells. HeLa or CaSki cells (1 × 10^7^ of each) were cultured for 72 h in the presence or absence of 20 ng/mL rh-TGF-β. (**A**) EVs obtained from the supernatants of CC cells cultured in the absence (HeLa/EVs or CaSki-EVs) or presence of rh-TGF-β (HeLa + TGF-β/EVs or CaSki + TGF-β/EVs) were characterized by flow cytometry using the violet side scatter configuration (Violet-SSC) and 130–880 nm fluorescent nanoparticles. The EV gate width was determined to be between 130 and 880 nm. Representative dot plots of EVs obtained from HeLa (**BI**) and CaSki (**CI**) cell supernatants and the percentages of HeLa/EVs, HeLa + TGF-β/EVs, CaSki-EVs, and CaSki + TGF-β/EVs, either small (sky blue) or medium-large (ultramarine blue), positive for CD39 (**BII**,**CII**) and CD73 (**BIII**,**CIII**), respectively, are shown. The percentages of total EVs (small and medium-large) among the HeLa/EVs, HeLa + TGF-β/EVs, CaSki/EVs, and CaSki + TGF-β/EVs positive for CD39 (**BIV**,**CIV**) or CD73 (**BVI**,**CVI**) are shown. The MFI of CD39 and CD73 expression in small and medium-large HeLa-EVs (**BV**,**BVII**) and CaSki-EVs (**CV**,**CVII**) was normalized to 1. The statistical significance was calculated using the one-way ANOVA followed by Sidak’s post hoc test for multiple comparisons. Representative data from three independent experiments ± SEM are shown. Significant differences are indicated as follows: * *p* < 0.05, ** *p* < 0.001, *** *p* < 0.0001.

Using the Violet-SSC and FSC parameters in flow cytometry and fluorescent nanospheres of different sizes (130, 220, 450, 880, and 1330 nm), the detection region of EVs (EV Gate) was determined to be between 130 and 880 nm (Figure 3A). Moreover, EVs were also defined by their size into small (130 nm, sky blue) and medium-large (>130 nm, ultramarine blue), as previously reported by Yokoi et al., 2023 [29]; Nagao et al., 2024 [30]; and Pérez et al., 2023 [31].

The percentages of small and medium-large EVs positive for CD39 or CD73 in the supernatants of cells cultured under baseline conditions or in the presence of rh-TGF-β were determined. The percentages of small and medium-large HeLa/EVs and CaSki/EVs positive for CD39/CD73 were 7.18%/0.43% and 0.82%/1.57% and 2.18%/1.98% and 2.0%/18.99%, respectively (Figure 3(BII,BIII,CII,CIII)). Interestingly, there was an increase in the percentages of small and medium-large HeLa + TGF-β/EVs and CaSki + TGF-β/EVs positive for the expression of CD39/CD73, 10.4%/0.49% and 1.13%/3.59%, and 1.88%/4.06% and 1.86/34.1%, respectively (Figure 3(BII,BIII,CII,CIII)). In fact, the percentages of the total EVs (small and medium-large) positive for CD39/CD73 were higher in the HeLa + TGF-β/EVs and CaSki + TGF-β/EVs than in the HeLa/EVs and CaSki/EVs, respectively (Figure 3BIV,BVI,CIV,CVI).

To evaluate the effect of TGF-β on the expression of CD39 and CD73 in EVs, the mean fluorescence intensity (MFI) of the small and medium-large HeLa/EVs or CaSki/EVs positive for CD39 or CD73 was normalized to 1. We observed that small HeLa + TGF-β/EVs (Figure 3B(V)), medium-large HeLa + TGF-β/EVs (Figure 3B(VII)), and small and medium-large CaSki + TGF-β/EVs (Figure 3C(VII)) presented significantly increased expression of CD39 or CD73, with MFI values of 1.20 ± 0.1 and 1.4 ± 0.221 and of 1.62 ± 0.08 and 1.6 ± 0.25, respectively. These results indicate that TGF-β increases the release of EVs enriched with CD39 and CD73 from CC cells.

**Figure 4:**
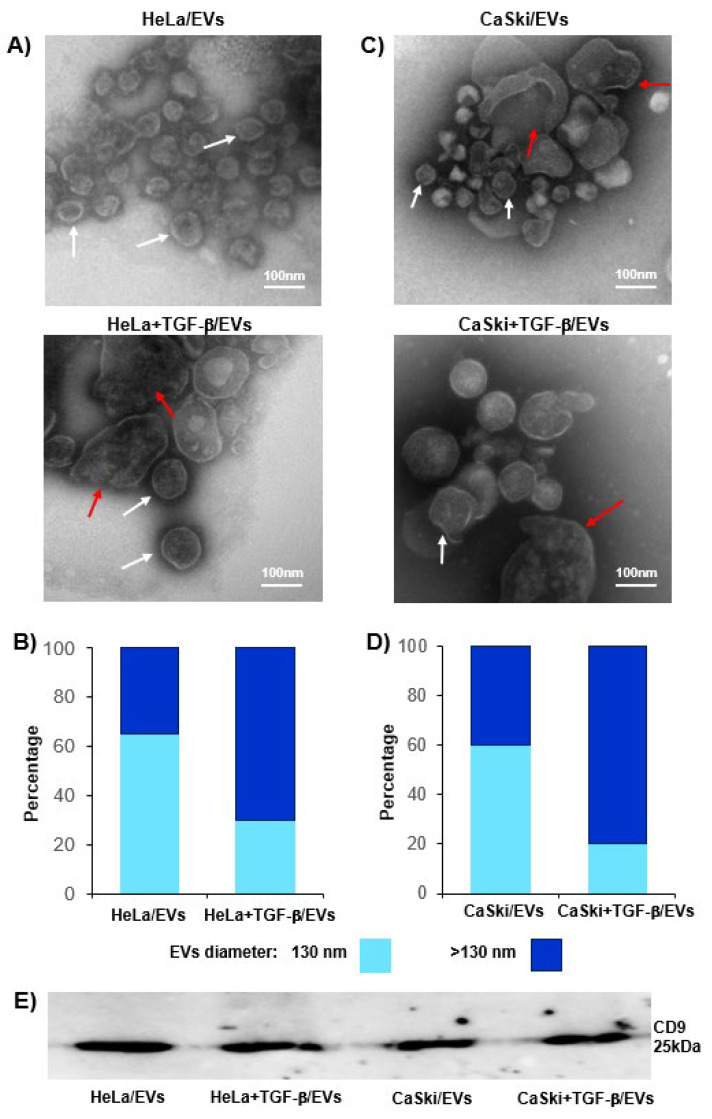
Characteristics of EVs released by CC cells cultured in the absence or presence of TGF-β. HeLa or CaSki cells (1 × 10^7^ of each) were cultured for 72 h in the presence or absence of 20 ng/mL rh-TGF-β. The EVs obtained from the supernatants of CC cells cultured in the absence (HeLa/EVs or CaSki-EVs) or presence of rh-TGF-β (HeLa + TGF-β/EVs or CaSki + TGF-β/EVs) were analyzed via transmission electron microscopy (**A**,**C**) and flow cytometry using fluorescent nanospheres of different sizes, as described in the Materials and Methods (**B**,**D**). The percentages of EVs with diameters < 130 nm (white arrows) and between 130 and 880 nm (red arrows) are also shown (**B**,**D**). The expression of CD9 in each of the EVs was determined by Western blotting, using twenty micrograms of total protein (based on the albumin type curve) (**E**).

Transmission electron microscopy revealed that HeLa + TGF-β/EVs and CaSki + TGF-β/EVs were larger than HeLa/EVs and CaSki/EVs, respectively (Figure 4A,C). Approximately 68% of the HeLa/EVs were 130 nm, whereas 72% of the HeLa + TGF-β/EVs were >130 nm (Figure 4B). On the other hand, 60% of the CaSki/EVs were 130 nm, whereas 85% of the CaSki + TGF-β/EVs were >130 nm (Figure 4D). Western blot analysis revealed that EVs obtained from the supernatants of HeLa and CaSki cells cultured in the presence or absence of rh-TGF-β were positive for the EV marker CD9 (Figure 4E).

### 2.4. EVs Were Secreted by TGF-β-Stimulated CC Cells and Had a High Capacity to Generate Ado

**Figure 5:**
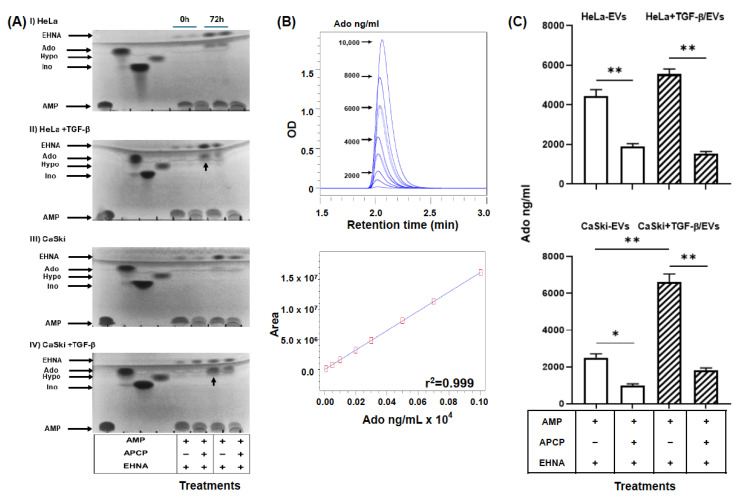
Adenosine is produced by EVs secreted by CC cells. EVs (50 μg) obtained from the supernatants of CC cells cultured in the absence (HeLa/EVs or CaSki-EVs) or presence (HeLa + TGF-β/EVs or CaSki + TGF-β/EVs) of 20 ng/mL rh-TGF-β were incubated for 72 h in the presence of 2.5 mM AMP, in the presence or absence of 2.5 mM APCP (an inhibitor of the enzymatic activity of CD73), or in the presence of 1 mM EHNA (an inhibitor of the enzymatic activity of adenosine deaminase). (**A**) The hydrolysis products were visualized via TLC. The increased capacity of HeLa + TGF-β/EVs or CaSki + TGF-β/EVs to hydrolyze AMP is indicated by arrows. (**B**) The amount of adenosine (Ado) produced by EVs was evaluated via UPLC using a typical Ado standard curve. (**C**) The concentrations of Ado generated by the EVs ± SEM are shown. The statistical significance was calculated using the one-way ANOVA followed by Sidak’s post hoc test for multiple comparisons. * *p* < 0.05; ** *p* < 0.01. Representative data from three independent experiments are shown. AMP, adenosine monophosphate; APCP, adenosine 5′-(α,β-methylene) diphosphate; EHNA, (+) erythro-9-(2-hydroxy-3-nonyl) adenine; Hypo, hypoxanthine; Ino, inosine.

To analyze the capacity of the EVs secreted by CC cells to generate Ado, 50 μg of HeLa/EVs, CaSki/EVs, HeLa + TGF-β/EVs or CaSki + TGF-β/EVs were incubated for 72 h in the presence of 2.5 mM AMP and in the presence or absence of 2.5 mM adenosine 5′-(α,β-methylene)diphosphate (APCP), an inhibitor of CD73 enzymatic activity. The hydrolysis products were analyzed by thin-layer chromatography (TLC). The different EVs were able to convert AMP nucleotides into inosine (Ino) and hypoxanthine (Hypo). However, the addition of APCP, an inhibitor of CD73 activity, only partially inhibited the generation of Ino by CC cells (Supplementary Figure S1), suggesting the presence of the enzymes adenosine deaminase (ADA), which converts Ado to Ino, and purine nucleoside phosphorylase (PNP), which converts Ino to Hypo, in EVs. To inhibit the deamination of Ado by ADA, 1 mM *erythro*-9-(2-hydroxy-3-nonyl) adenine (EHNA), an inhibitor of ADA activity, was added. The generation of Ado was detected by TLC (Figure 5A) and ultra-performance liquid chromatography (UPLC) (Figure 5B,C). Interestingly, HeLa + TGF-β/EVs and CaSki + TGF-β/EVs generated greater amounts of Ado than did HeLa/EVs and CaSki/EVs; the concentrations of Ado were 5550 ± 260 and 6600 ± 450 pg/mL and 4450 ± 320 and 2500 ± 210 pg/mL, respectively (Figure 5C). The addition of APCP strongly reduced the generation of Ado by HeLa + TGF-β/EVs, CaSki + TGF-β/EVs, HeLa-EVs and CaSki-EVs; the concentrations of Ado were 1528 ± 120 and 1800 ± 142 pg/mL and 1900 ± 150 and 1000 ± 89 pg/mL, respectively (Figure 5C).

## 3. Discussion

In recent years, several studies have provided evidence of the participation of EVs in the development of cancer through the transfer or direct action of the bioactive content released by EVs on recipient cells, thus affecting multiple biological processes within the TME, such as modifying the activity of stromal cells, activating drug resistance genes, promoting immune evasion and suppression of the immune response, generating mutations in target cells, promoting the migration and invasion of tumor cells and creating premetastatic niches, among other processes [32,33]. Although EVs derived from cancer cells are carriers of tumor antigens, which can in principle mediate antitumor immunity, a large body of evidence suggests that EVs act primarily to suppress the host immune response [34]. For example, Fas ligand (FasL), an apoptosis-inducing ligand associated with TNF (TRAIL), and programmed cell death ligand 1 (PD-L1), which are expressed on the surface of EVs secreted in the TME, can inhibit the proliferation and activation of CD8+ T cells [35,36]; for this reason, this topic needs more research and attention.

For CC and other types of HPV+ cancer, EVs secreted by tumor cells may contain different biomolecules, including the oncoproteins E6* and E7* and proteins that regulate the immune response, such as TGF-β, FasL, OX40, and OX40L [37]; microRNAs [38,39]; lncRNAs [40]; circRNAs [41]; mRNAs [42]; E1*, L1*, E6*, and E7* DNA [43]; and purine metabolites that promote immune escape [44]. However, little is known about the factors that regulate the secretion and loading of EVs released by CC cells. In recent years, there has been evidence that signaling through inflammatory pathways plays an important role in the regulation of EV biogenesis during the carcinogenesis process [45]. In this context, it has also been reported that TGF-β signaling promotes the release of EVs and that this is related to the regulation of several transcription factors, such as Snail, ZEB1, ZEB2, TWIST1, and TWIST2 [26]. Stromal cells, in addition to cancer cells, play an important role in tumor progression through the production of TGF-β in the TME [46]. In CC, the production of TGF-β is directly correlated with the degree of disease progression and the suppression of the antitumor immune response [15]. In fact, high levels of this factor have been detected in the tissues and sera of patients infected with HR-HPV and in patients with cervical intraepithelial neoplasia (CIN) and CC [45,46].

Our group provided evidence for the first time that the adenosinergic pathway plays an important role in the induction of the secretion and expression of TGF-β1 in CC tumor cells through the generation of Ado and the activation of A2AR and A2BR [17]. In fact, we also found that the levels of CD39 and CD73 in the cervical mucus or plasma of patients with HR-HPV infection correlated with high plasma levels of TGF-β and with the degree of disease progression [47,48,49], suggesting an important role for this factor in the release of both ectonucleotidases. To investigate the effects of TGF-β on the expression of CD39 and CD73 in EVs secreted by CC cells, in the present study, we cultured CaSki and HeLa cells in vitro in the presence of rh-TGF-β. We found that TGF-β is an important factor that induces and maintains the expression of CD39 and CD73 in these cells because the addition of SB505124, a selective inhibitor of the TGF-β1 receptor, to cultures of HeLa and CaSki cells significantly reduced the expression of CD39 and CD73. This result is supported by previous studies showing that CC cells in culture secrete significant amounts of TGF-β, which acts in an autocrine manner to maintain the expression of CD73 [17]. On the other hand, we also reported that CC tumor cells positive for HPV-16 and HPV-18 express higher levels of CD73 than do those negative for HPV and strongly suppress the effector functions of cytotoxic T lymphocytes through the production of Ado [47]. These results are in accordance with results recently reported by Cristian-Iser et al. (2025), which showed that CC cell lines positive for HPV presented higher CD73 expression than did HPV-negative cell lines [50]. In the present study, we observed that CaSki cells presented greater amounts of CD39/CD73 than did HeLa cells and that both cell lines were induced to increase the expression of CD39 and CD73 when cultured in the presence of TGF-β; however, we also observed that the C33A cell line, which is negative for HPV infection and has marginal CD73 expression [47], did not respond to the TGF-β stimulus to increase the expression of CD39 and CD73 (Supplementary Figure S2). These findings indicate that HPV infection is able to modulate CD39 and CD73 expression in CC cells. In fact, we previously reported that when the E6 and E7 oncogenes were silenced in HeLa cells, the expression level of CD73 and its ability to produce Ado were strongly reduced [47]. This phenomenon is likely associated with TGF-β1 production, since TGF-β1 expression in CC is positively correlated with the expression of HR-HPV oncogenes [51] and the HPV-16 oncogenes E6 and E7 induce activation of the human TGF-β1 promoter through the Sp1 recognition sequence [52]. Therefore, in subsequent studies, analyzing whether the HPV genotype or the status of integration influences the modulation of CD39 and CD73 expression in CC cells will be interesting.

The induction of CD39 and CD73 expression by rh-TGF-β in CC cells coincides with previous reports showing the role of this cytokine as a positive regulator of the expression of these ectonucleotidases in various cell types. For example, the expression of CD39 and CD73 in myeloid-derived suppressor cells (MDSCs) is induced through TGF-β-mTOR-HIF-1 signaling in patients with non-small cell lung cancer [53]. Similarly, TGF-β is important for the induction of CD73 expression in breast and prostate cancer cells [54,55], suggesting that other factors can influence the production of TGF-β1 to promote tumor growth. The mechanism by which TGF-β increases mRNA levels and the synthesis of these ectonucleotidases has not yet been fully elucidated. However, it has been proposed that TGF-β regulates CD73 through transcription factors such as SMAD and SP1 [56] and can also activate transcription factors such as HIF-1α or β-catenin/TCF-1, which bind to regulatory elements in the promoters of NT5E and ENTPD1 and increase the stability of coding mRNAs or prolong the half-life of proteins by preventing their degradation [57,58].

**Figure 6:**
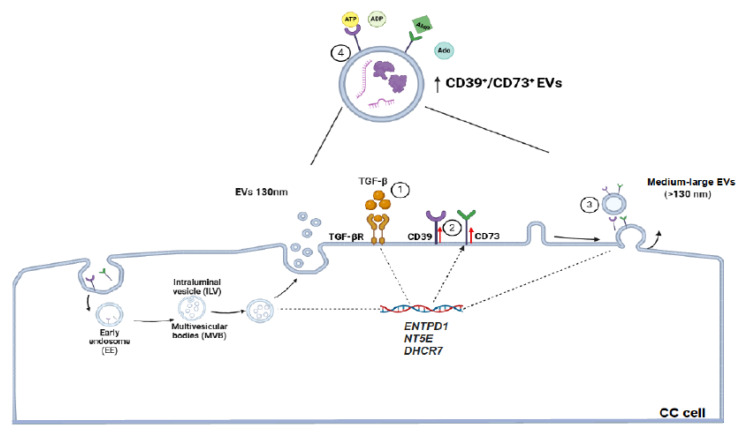
Proposed mechanism by which TGF-β1 induces the expression of CD39 and CD73 and promotes the secretion of medium-large EVs enriched with these ectonucleotidases from CC cells. The interaction of TGF-β1 with its receptor (TGF-βR) (1) induces (dotted lines) an increase in the expression of *ENTPD1* and *NT5E*, genes that encode CD39 and CD73, respectively, and probably the *DHCR7* gene (recently reported by Rodrigues-Junior et al. 2025 [63]), which encodes 7-dehydrocholesterol-reductase. The increase in the membrane expression of CD39 and CD73 (2), as well as the secretion of EVs promoted by cholesterol synthesis via 7-dehydrocholesterol-reductase activity, which can facilitate the biogenesis and secretion of medium-large active CD39- and CD73-enriched EVs (3), contributes to the generation of high Ado levels (4). Images were created in biorender.com.

We detected that TGF-β1 strongly induced the secretion of CD39/CD73 ectonucleotidases in the HeLa and CaSki cell supernatants. However, the addition of GW4869, a known inhibitor of exosome formation [59], strongly inhibited (>50%) the secretion of both ectonucleotidases, suggesting that EVs are responsible for the majority of ectonucleotidases released from CC cells stimulated with TGF-β. A decrease in the secretion of CD73-positive EVs has also been observed in T cells cultured in the presence of GW4869 [60]. For the characterization of EVs isolated from HeLa and CaSki cell supernatants, we used fluorescent nanospheres of different sizes, which allowed us to detect EVs between 130 and 880 nm via flow cytometry. Interestingly, we detected a higher percentage of CD39- and CD73-positive EVs with larger sizes in the supernatants of CC cells cultured in the presence of TGF-β (HeLa + TGF-β/EVs and CaSki + TGF-β/EVs) than in the supernatants of CC cells cultured in the absence of this factor (HeLa-EVs and CaSki-EVs). Similarly, the expression of CD39 and CD73 was greater in HeLa + TGF-β/EVs and CaSki + TGF-β/EVs than in HeLa-EVs and CaSki-EVs, respectively. The expression and functional activity of CD39 and CD73 in exosomes derived from different tumor cells have been reported in some studies [18,20,22,25]. However, in this study, we report for the first time that the stimulation of CC cells with TGF-β promoted the secretion of medium-large EVs (>130 nm) enriched with CD39 and CD73, which have a high capacity to generate Ado. These results are in accordance with several studies performed with different types of tumor cells, which reported that TGF-β can influence the cargo of molecules contained in EVs [61]. For example, the microRNA profiles of EVs and colorectal cancer tumor cells are altered as a result of deficient TGFBR2 expression [62]. Recently, it has been proposed that TGF-β signaling through the activation of MEK/ERK1/2 and the phosphorylation of SREBP2 (steroid regulatory element binding protein-2) participates in the induction of the expression of DHCR7, a gene involved in the generation of cholesterol, which facilitates the biogenesis and secretion of EVs [63]. There is also evidence that TGF-β can regulate EV biogenesis by influencing ESCRT-independent mechanisms involving specific lipids (cholesterol, ceramide, and phosphatidic acid) and transmembrane proteins, such as tetraspanins (CD9, CD63, CD81, and CD82), which regulate cargo sorting and membranous microdomains that are incorporated into the forming EVs [64]. Consequently, subsequent studies could evaluate whether TGF-β induces the expression of DHCR7 in CC cells and whether the cargo of CD39 and CD73 in EVs varies according to their biogenesis, which can be very useful biomarkers for determining the diagnosis and prognosis of CC [65,66]. The findings of the present study imply that the increase in the secretion of medium-large EVs enriched with CD39 and CD73 by CC cells stimulated with TGF-β can contribute significantly to increased adenosine levels in the TME and consequently inhibit the activation of T cells, even in the absence of direct interactions between T cells and EVs (Figure 6). 

The inhibition of the secretion of EVs and their cargo in cancer cells, has been recently proposed as a therapeutic approach in cancer [60,61]. In this context, several in vitro studies, and some in vivo preclinical studies, have indicated that many compounds have the ability to block, or at least limit, the formation and release of exosomes and/or microvesicles, these include imatinib, calpeptin, cytochalasin D, Y27632, glyburide, pantetin, imipramine, dimethylamiloride, GW4869, chloramidine, manumycin A, sulfisoxazole, bisindolylmaleimide I, clopidogrel, NSC23766, indomethacin and U0126 [60]. However, larger studies are needed to investigate the activities of these drugs, alone and in combination, to selectively block vesicles from diseased cells (such as cancer cells) rather than vesicles from normal healthy cells.

## 4. Materials and Methods

### 4.1. Cell Culture

The CC, CaSki (HPV-16+), and HeLa (HPV-18+) cell lines used in this study were obtained from the American Type Culture Collection (ATCC) and were free of mycoplasma. The authenticity of each line was verified via short tandem repeat (STR) genetic profiling, which was carried out 6 months before the start of the experiments. The cells were cultured on plates with RPMI 1640 medium (Sigma–Aldrich, St. Louis, MO, USA) supplemented with 10% fetal bovine serum (FBS; Gibco, Grand Island, NE, USA), 100 U/μL penicillin, 100 U/μL streptomycin (Gibco, San Diego, CA, USA), and 5 mM L-glutamine (Gibco, San Diego, CA, USA) and were incubated at 37 °C with 5% CO_2_ in an environment with saturated humidity.

### 4.2. Expression of CD39 and CD73

CC cells (2.5 × 10^5^) were cultured for 72 h in the presence or absence of 20 ng/mL rh-TGF-β (PeproTech, Inc., Rocky Hill, NJ, USA). In some assays, 25 µM SB505124 (Sigma–Aldrich, St. Louis, MO, USA), a selective inhibitor of TGF-β type I receptors, was added to block the effect of TGF-β. The expression of CD39 and CD73 in CC cells was assessed by flow cytometry using the monoclonal antibodies anti-CD39-FITC (BioLegend^®^, San Diego, CA, USA) and anti-CD73-PE (BD Biosciences, San Diego, CA, USA), according to protocols previously reported by our working group [67]. The analysis was performed by acquiring 10,000 events on a FACS ARIA cytometer (BD Biosciences, San José, CA, USA). The results were analyzed and are reported as the MFI±SEM compared with the baseline expression of CD39 or CD73 in CC cells, which was normalized to the value of 1. All the experiments were carried out in triplicate.

### 4.3. Isolation of EVs

EVs were obtained following the protocol previously reported by Montesinos et al. (2020) [68] and characterized according to the recommendations of MISEV 2023 [19]. Briefly, 1 × 10^7^ CC cells were grown on 100 mm culture dishes (Nest Biotechnology, Wuxi, China). After the medium was removed, the cells were washed three times with PBS and cultured for 72 h in Opti-MEM^®^ serum-free culture medium (Life Technologies, Grand Island, NY, USA) in the presence or absence of 20 ng/mL rh-TGF-β. The supernatants were collected and centrifuged at 500× *g* for 10 min to pellet the cells in suspension, subsequently filtered through 0.2-µm pore membranes (Millipore, Darmstadt, Germany), and centrifuged consecutively at 2000× *g* for 20 min and at 16,000× *g* for 60 min. The pellets were washed and resuspended in PBS for flow cytometry analysis using a CytoFLEX LX cytometer (Beckman Coulter, Brea, CA, USA) with a violet lateral scatter configuration (Violet-SSC). The resolution capacity of the equipment was configured using fluorescent nanospheres with sizes of 130, 220, 450, 880, and 1330 nm (Spherotech, Lake Forest, IL, USA). The selection of the EV region (EV gate) was determined using Violet-SSC and forward scatter (FSC) measurements. To determine the expression of CD39 and CD73, the EVs were incubated for 30 min at 4 °C with the respective coupled monoclonal antibodies. The EVs were subsequently washed with 1 mL of previously filtered PBS and stored in a refrigerator for a maximum of 24 h. The analysis was performed by acquiring 1 × 10^6^ EVs on a CytoFLEX LX cytometer. The data were analyzed with CytExpert 2.0 software. The results were analyzed and are reported as the MFI ± SEM. The baseline expression of CD39 or CD73 in EVs obtained from cultured CC cells in the absence of TGF-β was normalized to a value of 1.

### 4.4. Analysis of EVs

EV analysis was carried out by transmission electron microscopy following the protocol previously reported by García-Rocha et al., 2022 [69]. Briefly, a total of 20 µL of EVs were placed onto 400 mesh copper grids with a carbon-coated Formvar film and incubated for 5 min. Excess liquid was removed by blotting, and the grids were air-dried. Next, 20 µL of 2% phosphotungstic acid, pH 7.0, was placed on the grids, followed by blotting to remove excess liquid. The grids were examined under a JEOL 1010 transmission electron microscope operated at 100 kV. Digital images were acquired with a CCD-300RT MT 1 Hamamatsu camera (Hamamatsu Photonics K.K., Shimokanzo, Iwata City, Japan).

### 4.5. Western Blot Analysis

To determine the presence of the tetraspanin CD9 in EVs, we followed the protocol previously reported by Mora-García et al., 2017 [47]. The HeLa/EVs, CaSki-EVs, HeLa + TGF-β/EVs, or CaSki + TGF-β/EVs were treated with ice-cold lysis buffer [5 mM EDTA, 1% Triton X-100, 50 mM Tris (pH 7.4), 140 mM NaCl, 1 mM PMSF, 1 μM leupeptin, 1 mM NaF, 100 μM Na_3_VO_4_, 1% aprotinin, and 1 μM pepstatin]. The protein concentration (based on the albumin type curve) was determined using Bradford reagent (Sigma–Aldrich, St. Louis, MO, USA). To normalize equal amounts of the sample in the Western blot assay (in accordance with the recommendations of MISEV 2023 guidelines [19]), twenty micrograms of total protein from EVs was separated via SDS–PAGE on a 12% Bis-Tris gel (Invitrogen, Carlsbad, CA, USA) and transferred to a pure nitrocellulose membrane (Bio-Rad, Hercules, CA, USA). The membranes were blocked in Tris-buffered saline with 0.1% Tween 20 (TBST) and 2% bovine serum albumin overnight at 4 °C and then analyzed with a mouse anti-CD9 antibody (Novus Biologicals^®^, Centennial, CO, USA) at a dilution of 1:500 in a solution of TBST, followed by incubation with a horseradish peroxidase (HRP)-conjugated rabbit anti-mouse antibody (Sera-care^®^, Milford, MA, USA). Proteins were visualized using the Bio-Rad^®^ ChemiDoc MD Imaging System and Lab Touch Software 4.0. All figures show representative results of at least three independent experiments.

### 4.6. Detection and Quantification of CD39 and CD73

The concentrations of the CD39 and CD73 proteins in CC cell supernatants were measured via ELISA using the protocol previously reported by Muñóz-Godínez et al., 2020 [49]. GW4869, a known inhibitor of exosome formation, was added to CC cultures [56]. The data were interpolated into standard curves using different concentrations (1–10 ng/mL) of recombinant human CD39 and CD73 enzymes (rh-CD39 and rh-CD73, R&D Systems, Minneapolis, MN, USA) diluted in PBS. One hundred microliters of supernatant from each cell line was added to triplicate wells in ELISA/RIA 96-well flat bottom plates (Corning, New York, NY, USA). The plates were incubated for 1 h at 37 °C, incubated overnight at 4 °C, subsequently washed with a washing solution (TBS-Tween 0.1%), and then incubated with a blocking solution (2% bovine albumin *w*/*v* in TBS-0.1% Tween-20) for 2 h at 37 °C. After washing, an anti-CD73 antibody or anti-CD39 (Novus Biologicals, Centennial, CO, USA) was added at a dilution of 1:1000 in blocking solution and incubated for 2 h at 37 °C. The plates were washed six times and incubated with the secondary antibodies alkaline phosphatase-conjugated goat anti-human IgG (H + L) (Zymed, San Francisco, CA, USA) and alkaline phosphatase-conjugated goat anti-rabbit IgG (H + L) (Millipore, Darmstadt, Germany) at a 1:1000 dilution in blocking solution for 2 h at 37 °C. After eight washes, alkaline phosphatase substrate (Sigma–Aldrich, St. Louis, MO, USA) diluted in 10% diethanolamine (Sigma–Aldrich, St. Louis, MO, USA) was added (pH 9.8). Finally, the absorbance was measured at a wavelength of 405 nm in an ELISA plate reader.

### 4.7. Evaluation of CD73 Hydrolytic Activity in EVs

The ability of EVs secreted by CC cells to generate Ado was analyzed via TLC and UPLC. For this purpose, 50 μg of EVs were incubated for 72 h in the presence of 5 mM AMP in the presence or absence of 5 mM APCP, an inhibitor of CD73 enzymatic activity (Sigma–Aldrich, St. Louis, MO, USA), and in the presence or absence of EHNA (Sigma–Aldrich, USA), a specific ADA inhibitor, at a final concentration of 1 mM. The total volume of each reaction was 100 µL. For the analysis of AMP hydrolysis products, 1 µL of each supernatant was placed on plates coated with fluorescent gel (Whatman, GE Healthcare, Freiburg, Germany). The samples were eluted for 1 h using mobile phases composed of isobutanol:isoamyl alcohol:ethoxyethanol:ammonia:water (9:6:18:9:15) and isobutanol:ethyl acetate:methanol:ammonia (7:4:3:4) [64]. ATP, ADP, AMP, Ado, Ino, and Hypo (Sigma–Aldrich, St. Louis, MO, USA) were used at 5 mM as standard controls. The compounds were visualized using a UV transilluminator.

The amount of Ado generated in each sample incubated with AMP was evaluated via UPLC using the protocol previously reported by Mora-García et al., 2016 [67]. Briefly, 20 µL of each supernatant was eluted using a chromatograph (UPLC ACQUITY, Waters Corporation, Milford, MA, USA) with a mobile phase composed of 0.5% acetonitrile, 5% methanol, and 94.5% sodium acetate (0.25 M and pH 6.3). Before reading, the samples were filtered through Amicon 3000D filters (Millipore). An Ado standard curve was prepared with Empower 3 (Waters Corporation, Milford, MA, USA) to evaluate the Ado concentrations in the different samples. The run conditions were as follows: a flow rate of 1.0 mL/min, UV detection at 254–260 nm, a retention time of 2.0 min, room temperature, and a LiChrospher 5 μm RP-18e 100 A (size 125 mm Å~4 mm, 5 μm particle size) reversed-phase column. Ado was quantified by comparing the retention time of the sample with that of the synthetic Ado used as a standard.

### 4.8. Statistical Analysis

The data are presented as the average of three experiments performed in triplicate, and the values are presented as the means ± SEMs of the data obtained. Statistical analysis was performed with GraphPad Prism 8.0 software via one-way ANOVA followed by Sidak’s or Dunnett’s post hoc test for multiple comparisons. *p* < 0.05 indicated statistical significance.

## 5. Conclusions

This study provides evidence, for the first time, that TGF-β induces an increase in the expression of CD39 and CD73 in CC cells and promotes the secretion of medium-large EVs enriched with these ectonucleotidases with a high capacity to generate Ado. These results suggest that TGF-β, a factor highly expressed in the CC TME, may favor neoplastic progression by increasing adenosinergic activity mediated by tumor cells and the secretion of active CD39- and CD73-enriched EVs. Consequently, the inhibition of CD39+ CD73+ EVs could be a strategy for the treatment of CC.

## Data Availability

All the data displayed in this publication, including the Supporting Information, are available from the corresponding author upon request.

## Supplementary Materials

The following supporting information can be downloaded at https://www.mdpi.com/article/10.3390/ijms26062413/s1.

## Abbreviations

The following abbreviations are used in this manuscript:

Ado	adenosine
ADA	Adenosine deaminase
ADP	adenosine diphosphate
AMP	adenosine monophosphate
APCP	adenosine 5′-(α,β-methylene)diphosphate
ARs	adenosine receptors
ATP	adenosine triphosphate
CC	cervical cancer
CD39	ectonucleoside triphosphate diphosphohydrolase-1
CD73	5′-ectonucleotidase
EHNA	*erythro*-9-(2-hydroxy-3-nonyl) adenine
ELISA	enzyme-linked immunosorbent assay
EVs	extracellular vesicles
HR-HPV	high-risk human papillomavirus
MFI	mean fluorescence intensity
MRS1754	A_2B_R antagonist
PD-1	programmed cell death-1
PD-L1	programmed death-ligand 1
TGF-β1	transforming growth factor-beta 1
TLC	thin-layer chromatography
TME	tumor microenvironment
UPLC	ultra-performance liquid chromatography
ZM241385	A_2A_R antagonist
