# Supplementary material for: TGF-β Induces the Secretion of Extracellular Vesicles Enriched with CD39 and CD73 from Cervical Cancer Cells
Source: Int J Mol Sci. 2025 Mar 7;26(6):2413. doi: 10.3390/ijms26062413 (PMC11942456; doi:10.3390/ijms26062413)
Supplement: Supplementary file 1 [file ijms-26-02413-s001.zip › ijms-3415480-supplementary.pptx]

## Slide 1
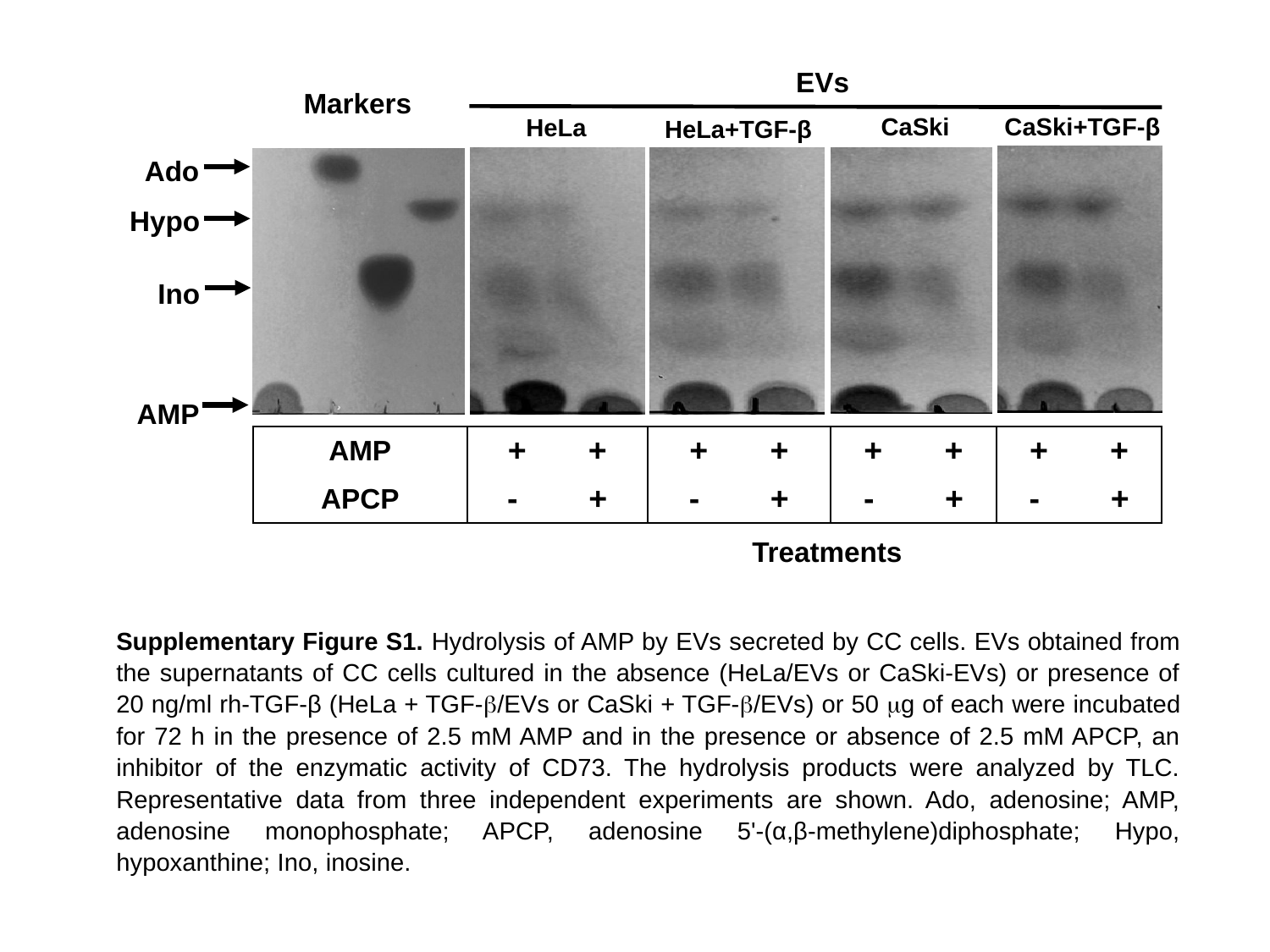

EVs
Markers
CaSki+TGF-β
CaSki
HeLa
HeLa+TGF-β
Ado
Hypo
Ino
AMP
| AMP | + + | + + | + + | + + |
| --- | --- | --- | --- | --- |
| APCP | - + | - + | - + | - + |
Treatments
Supplementary Figure S1. Hydrolysis of AMP by EVs secreted by CC cells. EVs obtained from the supernatants of CC cells cultured in the absence (HeLa/EVs or CaSki-EVs) or presence of 20 ng/ml rh-TGF-β (HeLa + TGF-β/EVs or CaSki + TGF-β/EVs) or 50 μg of each were incubated for 72 h in the presence of 2.5 mM AMP and in the presence or absence of 2.5 mM APCP, an inhibitor of the enzymatic activity of CD73. The hydrolysis products were analyzed by TLC. Representative data from three independent experiments are shown. Ado, adenosine; AMP, adenosine monophosphate; APCP, adenosine 5'-(α,β-methylene)diphosphate; Hypo, hypoxanthine; Ino, inosine.

## Slide 2
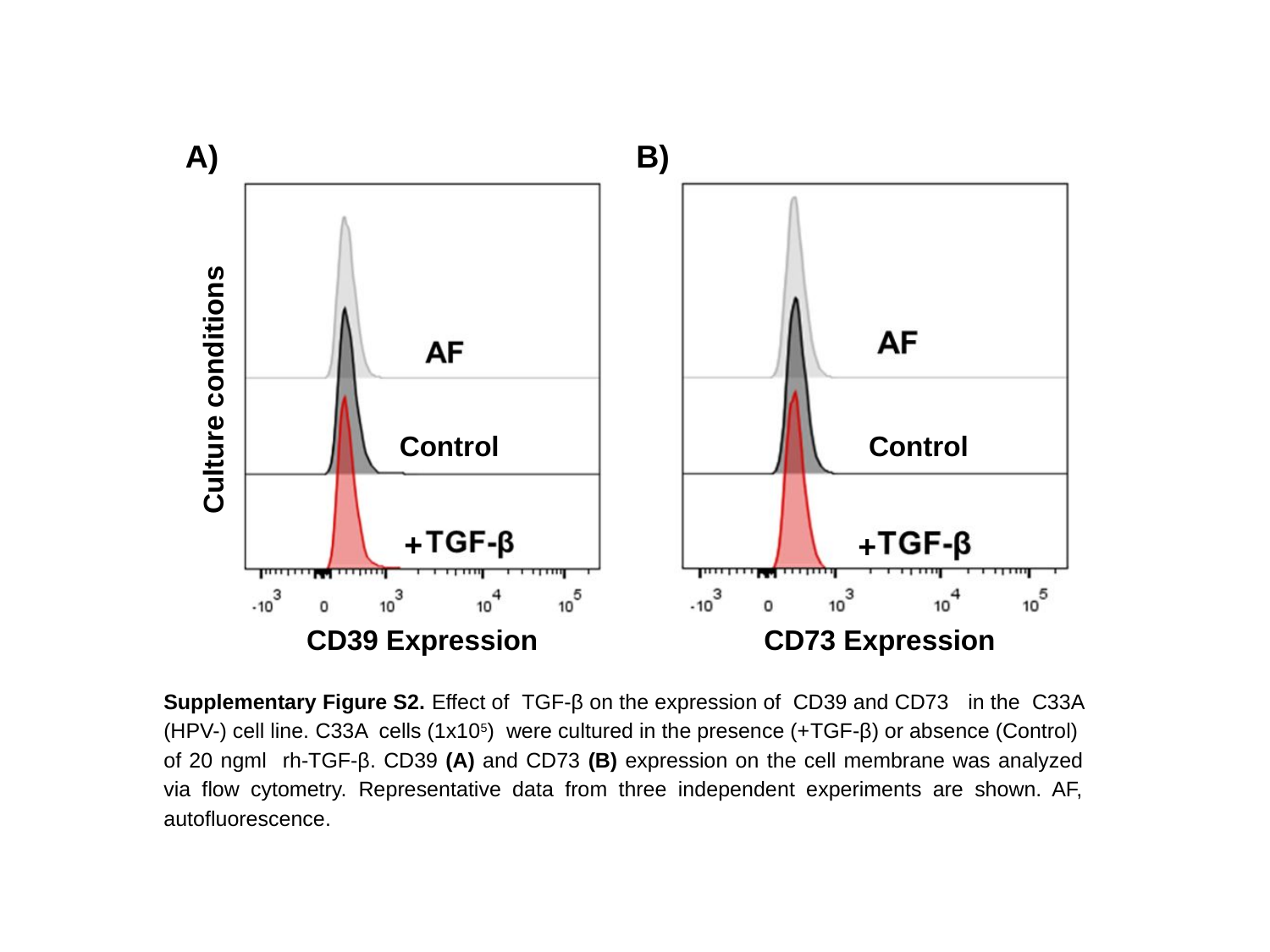

B)
A)
Culture conditions
Control
Control
+
+
CD39 Expression
CD73 Expression
Supplementary Figure S2. Effect of TGF-β on the expression of CD39 and CD73 in the C33A (HPV-) cell line. C33A cells (1x10^5^) were cultured in the presence (+TGF-β) or absence (Control) of 20 ngml rh-TGF-β. CD39 (A) and CD73 (B) expression on the cell membrane was analyzed via flow cytometry. Representative data from three independent experiments are shown. AF, autofluorescence.
